# Different Involvement of Promoter Methylation in the Expression of Organic Cation/Carnitine Transporter 2 (OCTN2) in Cancer Cell Lines

**DOI:** 10.1371/journal.pone.0076474

**Published:** 2013-10-16

**Authors:** Qiang Qu, Jian Qu, Min Zhan, Lan-Xiang Wu, Yi-Wen Zhang, Xiao-Ya Lou, Li-Juan Fu, Hong-Hao Zhou

**Affiliations:** 1 Institute of Clinical Pharmacology, Hunan Key Laboratory of Pharmacogenetics, Xiangya School of Medicine, Central South University, Changsha, Hunan, China; 2 Institute of Life Sciences, Chongqing Medical University, Chongqing, China; University of Hyderabad, India

## Abstract

Organic cation/carnitine transporter 2 (OCTN2) is responsible for the cellular uptake of the antineoplastic agent, oxaliplatin. Epigenetic modification is a possible mechanism of altered drug-transporter expression in cancers, leading to altered efficacy of chemotherapeutic drugs. However, the mechanisms governing OCTN2 regulation are not completely understood. In this study, the low levels of OCTN2 in HepG2 and LS174T cells were elevated by the demethylating reagent, decitabine (DCA). To further reveal the epigenetic mechanism of down-regulation of OCTN2, we found that Region-1 within the *OCTN2* promoter (spanning −354 to +85) was a determinant of OCTN2 expression in a luciferase reporter assay. Moreover, methylation-specific PCR (MSP) and bisulfite genomic sequencing showed that the degree of individual methylated CpG sites within this region was inversely correlated with the levels of OCTN2 in different cancer cells. Application of DCA to HepG2 and LS174T cells reversed the hypermethylation status of the *OCTN2* promoter and increased OCTN2 expression, enhancing cellular uptake of oxaliplatin. Thus, we identified that promoter methylation is responsible for epigenetic down-regulation of OCTN2 in HepG2 and LS174T cells. Given the essential role of OCTN2 in cancer cell uptake of chemotherapeutics, and thus treatment efficacy, pretreatment with a demethylating reagent is a possible strategy for optimizing pharmacotherapies against cancers.

## Introduction

The human *SLC22A5* gene, which encodes a 63 kDa organic cation/carnitine transporter 2 (OCTN2), is located in the cytokine cluster region on chromosome 5q31 [1], [2]. OCTN2 is expressed in various tissues, including kidney, skeletal muscle, heart, colon, brain, liver, etc [3]. Functional defects of OCTN2 are associated with various diseases including primary carnitine deficiency, Crohn's disease, and asthma [4]–[8]. OCTN2 not only transports carnitine, but also recognizes clinically important therapeutics such as mildronate, verapamil, pyrilamine, oxaliplatin, imatinib and cephaloridine [9]–[13]. OCTN2 is associated with oxaliplatin accumulation and cytotoxicity in OCTN2-HEK293 transfected cells [11]. The two alleles of *OCTN2* (rs2631367 and rs2631372) may be important predictors in gastrointestinal stromal tumor patients receiving imatinib therapy [12]. These reports indicate that the functional defects and/or aberrant expression of OCTN2 may affect the disposition and subsequent therapeutic efficacy of its substrates.

Several reports suggest the involvement of peroxisome proliferator-activated receptor alpha (PPARA) and gamma (PPARG) in the transcriptional regulation of OCTN2 in various tissues. However, down-regulation of OCTN2 has been reported in tumors with high expression of PPARA and PPARG [14]–[16]. A recent study found that the decreased levels of OCTN2 in several epithelial cancer cell lines could be restored by the demethylating reagent 5-aza-cytidine [17]. These findings imply that other machineries cooperate with the transcription factor network to modulate the expression of OCTN2, such as DNA methylation.

DNA methylation is an important epigenetic mechanism that modulates gene expression. The CpG dinucleotide near transcriptional start sites is abundant in gene promoters, and is referred to as CpG islands. The methylation of CpG islands is associated with repressed gene transcription and abnormal DNA methylation can lead to aberrant gene expression. Unlike gene mutation, DNA methylation can be reversibly altered by demethylating agents such as decitabine (5-aza-2′-deoxycytidine, DCA) and 5-aza-citidine. These agents are incorporated into the DNA and inactivate DNA cytosine C5-methyltransferases [18]. Thus, we hypothesized that the differential methylation status of *OCTN2* may be correlated with the aberrant expression of OCTN2 in cancer cells.

In this study, we investigated whether the methylation of CpG islands acts as a possible mechanism responsible for the down-regulation of OCTN2 in cancer cell lines. By using methylation-specific PCR (MSP), bisulfite genomic sequencing, and *in vitro* methylation assays, we have provided evidence that promoter DNA methylation is an essential mechanism suppressing OCTN2 expression in cancer cell lines. Application of a demethylating reagent, which modulated the methylation status of the *OCTN2* promoter, increased the expression of OCTN2 and made cancer cells more sensitive to oxaliplatin.

## Materials and Methods

### Chemicals and Reagents

Decitabine, sodium bisulfate, hydroquinone and oxaliplatin were purchased from Sigma-Aldrich (St. Louis, MO). TRIzol reagent and Lipofectamine 2000 were obtained from Invitrogen (Carlsbad, CA).

### Cell Culture and Treatment with DCA

The hepatoma cell line HepG2, colon cancer cell line LS174T, glioma cell line U251, bile duct cancer cell line QBC-939 and African green monkey kidney cell line COS-7 were obtained from American Type Culture Collection (Manassas, VA). Cell lines were cultured in DMEM supplemented with 10% fetal bovine serum (FBS) at 37°C in a humidified incubator containing 5% CO_2_, except the QBC-939 cell line, which was cultured in RPMI 1640. Cells were treated with DCA at a final concentration of 0.5 μM or 1 μM and renewed every 24 h for one week.

### RNA purification, cDNA Synthesis and Quantitative Real-time PCR

Total cellular RNA was isolated from the treated cells using TRIzol reagent. cDNA was synthesized from 1 μg of total RNA using the first cDNA synthesis kit (Takara, Dalian). Quantitative RT-PCR was performed using SYBR Green (Bio-rad) and the primers are listed in Table S1. RT-PCR was performed over 40 cycles at 95°C for 30 s, at 60°C for 30 s, and 72°C for 30 s followed by a final extension at 72°C for 2 min. The relative mRNA expression was normalized to the housekeeping gene *β-actin* and the analysis was carried out using the 2^−ΔΔCt^ method [19].

### Western Blotting

Total cellular proteins were extracted and the concentrations were quantified by the BCA protein assay (Thermo Scientific, Rockford, IL). Proteins were subjected to SDS-polyacrylamide gel electrophoresis on 10% polyacrylamide gels and transferred to PVDF membrane. The membrane was probed with either an anti-rabbit OCTN2 antibody (1∶400, Abcam Labs, Cambridge, MA) or β-actin antibody (1∶1000, Abcam Labs). Subsequently, membranes were incubated with DyLight 800-labeled rabbit polyclonal antibody (1∶7500, KPL Inc, Gaithersburg, MD), protected from the light. The stained membranes were scanned by Odyssey Infrared Imaging System (LI-COR Biosciences). The ratios of OCTN2 against β-actin were calculated.

### Cell Viability Assay

Cancer cell lines were treated with 1 μM DCA or 0.1% DMSO for one week. Subsequently, cells were seeded into 96-well plates at a density of 2×10^4^/well, and exposed to oxaliplatin at various concentrations (0, 0.3, 1, 3, 10, 33, 100 or 330 μM) for 48 h. Cell viability was measured by the MTS assay (Promega, Madison, WI) and IC_50_ values were calculated using SPSS 13.0 version.

### Methylation-Specific PCR (MSP) and Bisulfite-Sequencing PCR (BSP)

To predict regions rich in CpG dinucleotides close to the *OCTN2* transcriptional start site, we used Methyl-Primer software (http://www.urogene.org/methprimer/) according to the following criteria: the length of CpG island >100 bp, observed/expected CpG ratio >0.6 and percentage of G plus C>50%. We searched the *OCTN2* genomic sequence including 1.5 kb upstream and 2 kb downstream of transcriptional start site (TSS). According to the predicted CpG islands, MSP and BSP primers were designed and are listed in Table S1. Genomic DNA of cancer cell lines was isolated and DNA bisulfite modification was performed as described [20]. Bisulfite modified DNA was recovered by Wizard DNA Clean Up System (Promega) and treated with 5.5 μl of NaOH then precipitated with ethanol. Precipitated DNA was re-suspended in water, and then amplified with MSP or BSP primers. The PCR condition was as follows: 94°C for 5 minutes, followed by 40 cycles of 94°C for 30 s, annealing temperature (Table S1) for 30 s, 72°C for 60 s, and finally 72°C for 5 min. The amplified DNA fragments were run on 2% agarose gels and stained with ethidium bromide. BSP products were purified by a DNA purification kit (Takara, Dalian) and inserted into pGEM-T easy vector (Promega). Five clones for every cell line were randomly selected and sequenced. The methylated sequence of *OCTN2* was checked for alignment using MethBLAST.

### Plasmid Construction and *in vitro* Methylation Assay

According to the predicted CpG islands in the genomic sequence, we divided the promoter into three regions. Three regions containing different CpG islands were amplified (Primers listed in Table S2) and inserted into the pGL4.17 vector (Promega). These recombinants, pGL4.17-Region1, Region2 and Region3 were verified by automated sequencing. The respective plasmid was linearized by *Xho* I and subjected to *in vitro* the methylation assay as described in our previous work [21]. The methylation products were then digested by *Kpn* I and inserted into the pGL4.17 vector. These new recombinants containing the methylated promoter regions were named pGL4.17-mRegion 1, pGL4.17-mRegion 2 and pGL4.17-mRegion 3, respectively.

### Transient Transfection and Luciferase Report Assay

COS-7 cells were seeded in 24-well plates at a density of 10^5^/well in 500 μl DMEM and 10% FBS. After 8 h, cells were transfected with constructed luciferase vectors (0.2 μg/well) using Lipofectamine 2000. The pGL4.17 vector lacking a promoter served as the negative control. Internal control vector pRL-TK (0.02 μg/well) was used to normalize luciferase activity. After 8 h, each well of transfected cells was washed twice then 100 μl of reporter lysis buffer was added to lyse cells. Each group had triplicate wells and was repeated more than three times. Dual luciferase activity (Relative light units, RLU) was determined by a luminometer.

### Statistical Analysis

All data were expressed as mean ± standard deviation. Statistical analyses were performed with a one-way ANOVA for significance followed by a post-hoc test using SPSS 13.0 software. Data comparisons with a *p* value of less than 0.05 (*p*<0.05) were considered significantly different.

## Results

### OCTN2 mRNA and Protein Levels in Different Cancer Cell Lines

**Figure 1:**
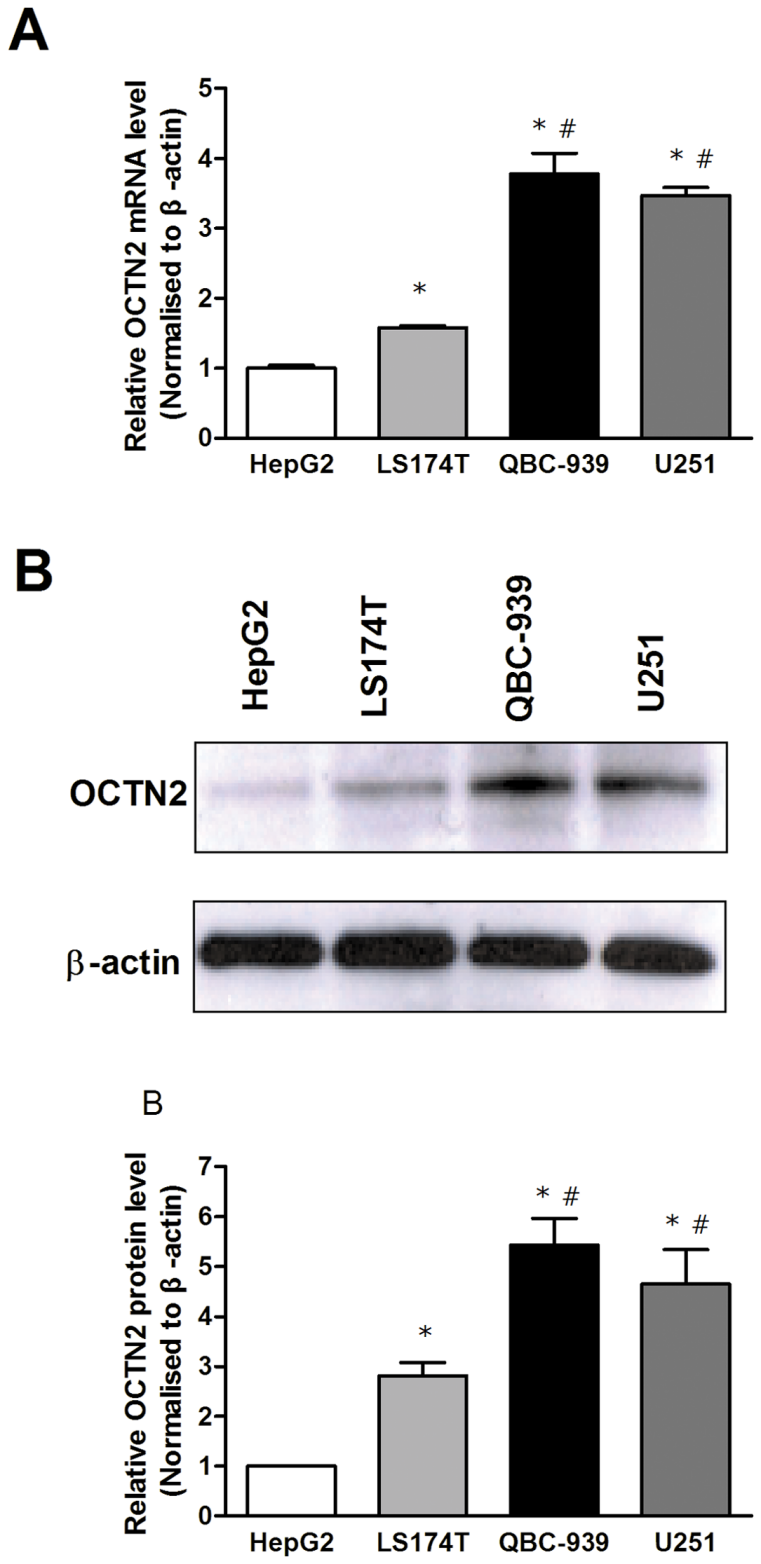
Expression of OCTN2 in cancer cell lines. (A), analysis of mRNA levels of OCTN2 in cancer cell lines. Total RNA in cancer cells was purified by TRIzol reagent for cDNA synthesis and the mRNA levels of OCTN2 were analyzed by Quantitative RT-PCR. (B), analysis of protein levels of OCTN2 in cancer cells. Total proteins were extracted and protein levels of OCTN2 were analyzed by western blotting. The relative expression of mRNA and protein was normalized to β-actin. The expression of HepG2 was set as 1. All of mRNA and protein analysis were performed three times. Significant difference from HepG2 or LS174T cell is denoted with asterisk (*, *p*<0.05) or number sign (#, *p*<0.05), respectively.

Total cellular RNA and protein were extracted from cancer cell lines and OCTN2 expression was measured by Quantitative RT-PCR and Western blotting, respectively. The highest expression of OCTN2 was in QBC-939, followed by U251, LS174T and HepG2 cells (Fig. 1). The expressions of OCTN2 in QBC-939 and U251 cells were significantly higher than that in U251, LS174T cells (*p*<0.05).

### Effects of DCA on the Expression of OCTN2 in Cancer Cell Lines

**Figure 2:**
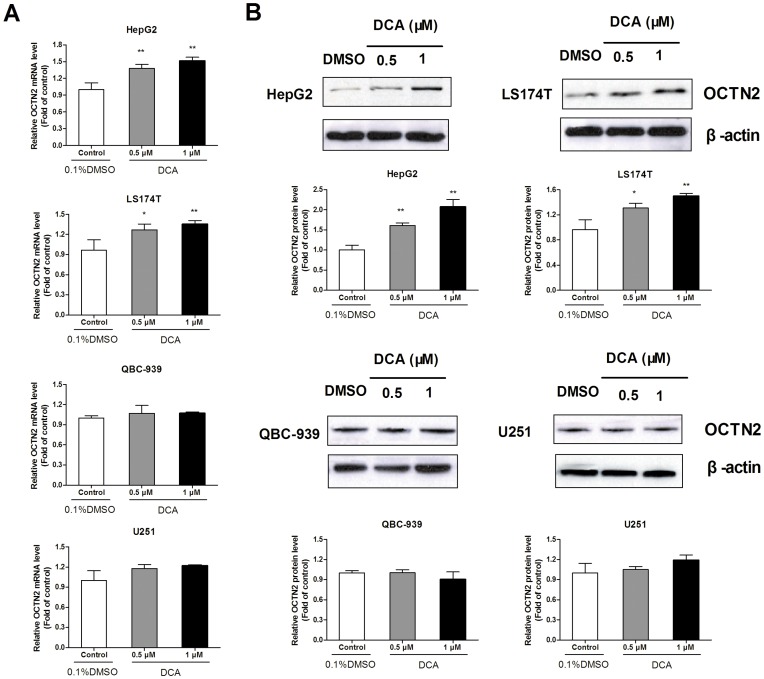
Effects of DCA on mRNA and protein levels of OCTN2 in cancer cell lines. (A), analysis of mRNA levels of OCTN2 in DCA-treated cancer cell lines. After treatment with 0.5 and 1 μM DCA, total RNA in cancer cells was purified by TRIzol reagent for cDNA synthesis and the mRNA levels of OCTN2 were analyzed by Quantitative RT-PCR. (B), analysis of protein levels of OCTN2 in DCA-treated cancer cell lines. Total proteins were extracted and protein levels of OCTN2 were analyzed by western blotting. The relative expression of mRNA and protein was normalized to β-actin. Data presented represent the mean ± S.D. of three independent experiments. Significant difference from control group of 0.1% DMSO is denoted with asterisks (*, *p*<0.05; **, *p*<0.01).

To examine whether the discrepant expressions of OCTN2 in different cancer cell lines was due to promoter methylation, we treated cancer cell lines with DCA and determined the restored expression of OCTN2. In Fig. 2, DCA remarkably up-regulated the relative OCTN2 mRNA and protein expression in HepG2 cells with 0.5 μM (1.38-fold, 1.61-fold) and 1 μM treatment (1.52-fold, 2.07-fold) (*p*<0.05), compared to the control group. Similarly, treatment of DCA lead to an increase of OCTN2 levels in LS174T cells. However, DCA did not change the expression of OCTN2 in QBC-939 and U251 cells, which already had high OCTN2 expression levels.

### OCTN2 Genomic Sequence Harbors Three CpG Islands

**Figure 3:**
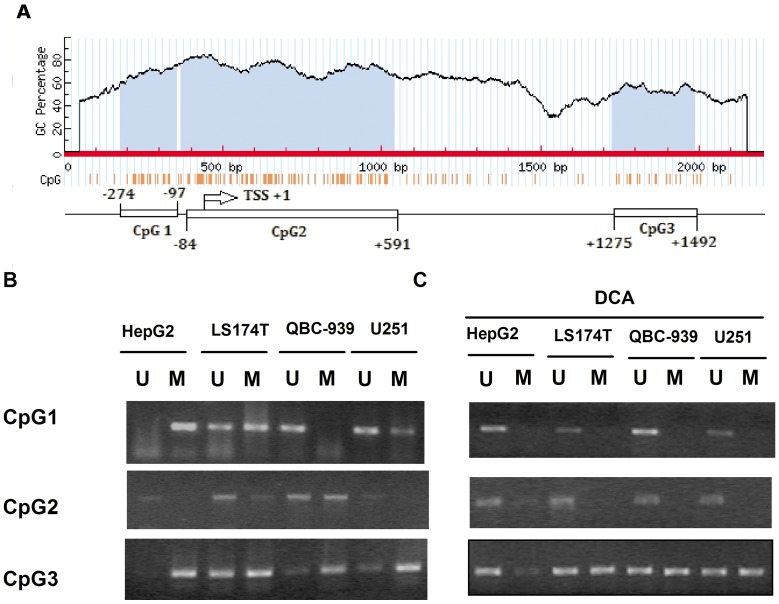
Methylation status of CpG islands was analyzed by MSP. (A), computational analysis revealed three putative CpG islands within the OCTN2 genomic sequence by Methyl-primer software as described in *Material and Methods*. (B), MSP analysis for CpG islands of OCTN2 in 1 μM DCA treated and non-treated cancer cell lines. MSP was performed with the specific primers to amplify the target CpG islands fractions and run on a 2% agarose gel. All of MSP and BSP analysis were repeated at three times. U: unmethylated sequence; M: methylated sequence.

We searched the *OCTN2* genomic sequence for the location of CpG dinucleotides using Methy-primer software. CpG dinucleotides were found extensively located near the TSS (Fig. 3A). According to the location intensity of CpG dinucleotides, we observed three putative CpG islands located in the *OCTN2* genomic sequence. CpG island-2 is located close to UTR and exon 1, CpG island-3 is located in intron 1, and CpG island-1 is located upstream of the TSS (Fig. 3A).

### Methylation Status of CpG Islands within OCTN2 by MSP

To further elucidate the methylation status of CpG islands within *OCTN2* in different cancer cell lines, we analyzed three CpG islands by MSP which distinguishes methylated DNA from un-methylated DNA. In Fig. 3B, CpG island-1 (CpG1) was completely methylated in HepG2 cells, partially methylated in LS174T and U251 cells, and un-methylated in QBC-939 cells. Partially methylated products of CpG island-2 (CpG2) were found in LS174T and QBC-939 cells, but these were un-methylated in HepG2 and U251 cells. In almost all cell lines, CpG island-3 had more methylated products than unmethylated. In Fig. 3C, after treatment with DCA, un-methylated products of CpG1 were increased in HepG2, LS174T and U251 cells. This finding indicates that DCA can cause de-methylation within CpG1. A similar result was observed within CpG2. DCA treatment increased un-methylated products of CpG3 in HepG2 cells, but did not cause significant changes in other cells.

### Effect of Methylated and Un-methylated Promoter Regions on Transcription Activity of OCTN2 using an *in vitro* Luciferase Reporter Assay

**Figure 4:**
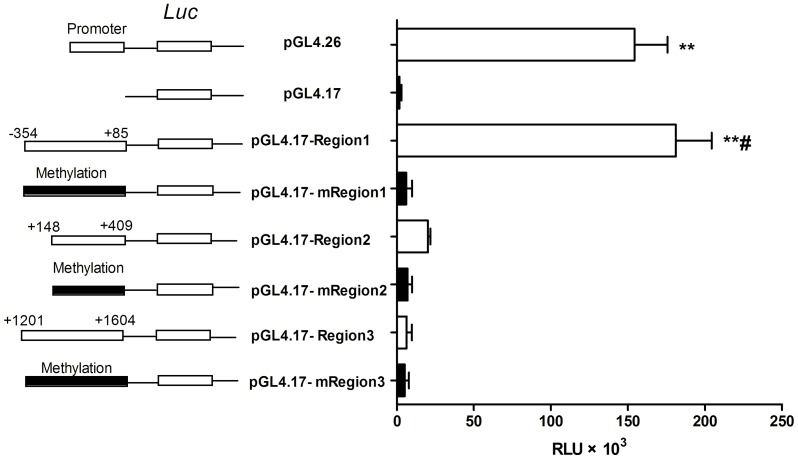
Methylation effects on transcriptional activity of the OCTN2 promoter in a luciferase reporter assay. Constructed pGL4.17 luciferase vector containing unmethylated and methylated promoter regions were transfected into COS-7 cells with the pRL-TK control vector. Forty-eight h later, the cells were washed and assayed for luciferase activity. Relative luciferase activity was measured in triplicate wells. Data presented represent the mean ± S.D. of three independent experiments. Significant difference from the treatment group of pGL4.17 is denoted with asterisks (*, *p*<0.05; **, *p*<0.01). Significant difference from the group of methylated region in the same region is denoted with a pound sign (#, *p*<0.05).

To determine which methylated CpG islands play essential roles in down-regulation of OCTN2, we constructed luciferase reporter plasmids bearing either un-methylated or methylated regions (Fig. 4). Constructed plasmids were transfected into COS-7 cells and used to assess transcriptional activity. In comparison with pGL4.17 (Control), pGL4.17-Region1 displayed markedly increased luciferase activity of 102.5-fold (*p*<0.01), while luciferase activity from pGL4.17-Region2 and Region3 were only increased 11.4-fold and 3.5-fold (*p*>0.05). This result indicates that Region1 spanning −354 to +85 bp plays an essential role in promoter activity. Interestingly, only the luciferase activity of un-methylated Region-1 was higher than its methylated vector (29.7-fold, *p*<0.01). Un-methylated Regions-2 and -3 did not show significant differences in luciferase activity compared to their corresponding methylated regions. It is possible that the methylation statuses of Region-2 and -3 containing CpG island-2 and -3, respectively, are irrelevant to *OCTN2* expression. Thus, these findings demonstrate that increased methylation can inhibit promoter activity of Region-1.

### Determination of DNA Methylation Profiles of OCTN2 by BSP

**Figure 5:**
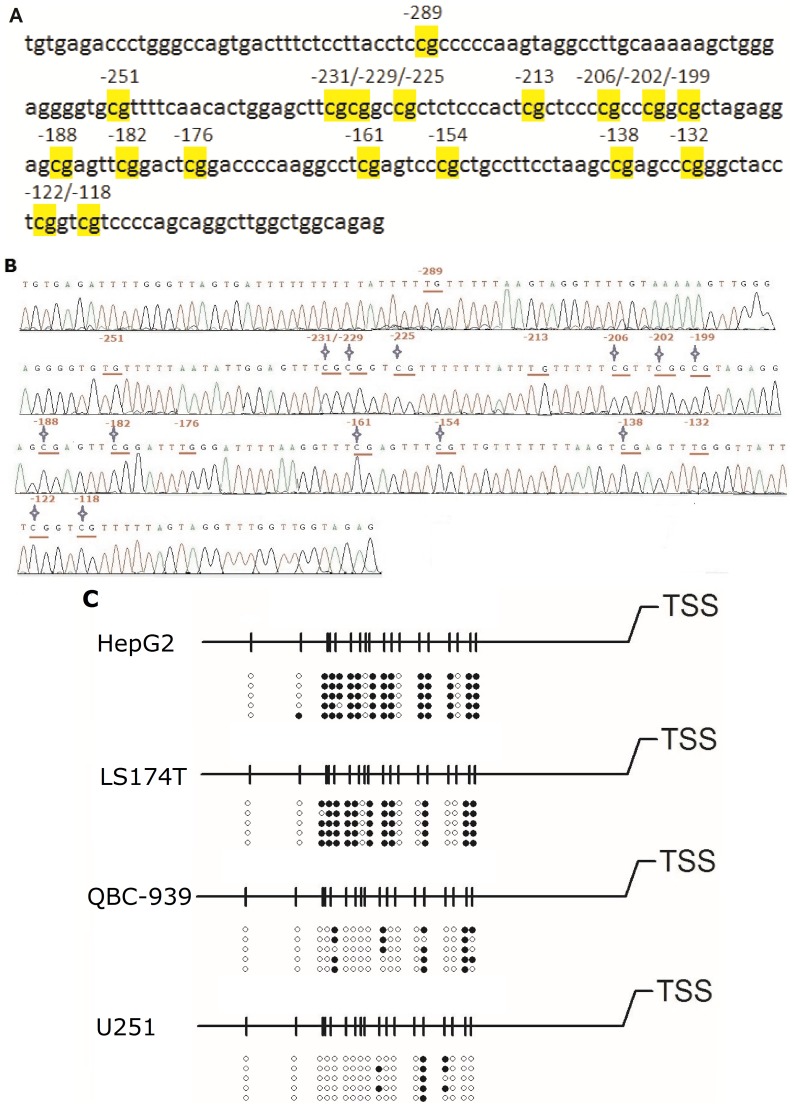
Methylation profiles around the transcriptional start site of *OCTN2*. (A), the genomic sequence spanning −325 to −92 bp in promoter Region-1 of *OCTN2*. Eighteen CpG sites were located and marked. (B), bisulfite genomic sequencing analysis of Region-1 of *OCTN2* in HepG2 cells. Bisulfite modified DNA was amplified by BSP as described under *Materials and methods* and then sequenced. Pointed star represents methylated CpG sites. (C), DNA methylation profiles of individual methylated CpG dinucleotides in four cancer cell lines. After BSP, five clones were randomly selected and sequenced. The methylated sequences were checked for alignment using MethBLAST. The open and closed circles represent unmethylated or methylated cytosines, respectively.

**Figure 6:**
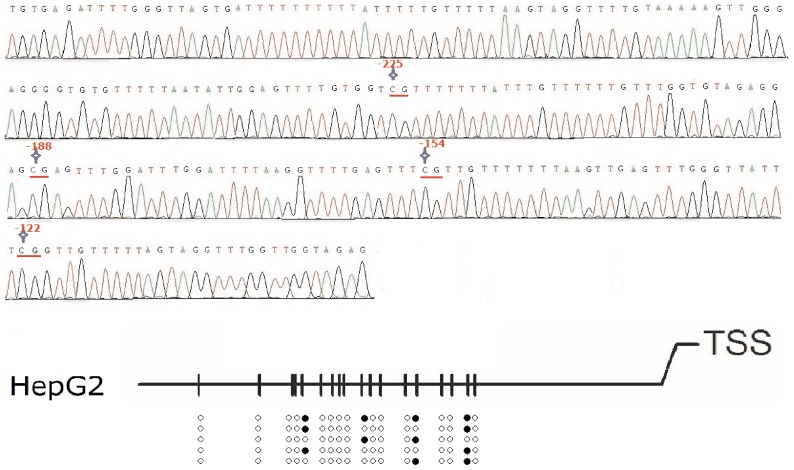
Methylation profiles of *OCTN2* in DCA-treated HepG2 cells. (A), bisulfite genomic sequencing analysis of DCA-treated HepG2 cells. After the treatment of DCA, DNA was extracted and subjected to bisulfite modification. Bisulfite modified DNA was amplified by BSP as described under *Materials and methods* and then sequenced. Pointed star represents methylated CpG sites. (B), DNA methylation profiles of individual methylated CpG dinucleotides in DCA-treated HepG2 cells. After BSP, five clones were randomly selected and sequenced. The methylated sequences were checked for alignment using MethBLAST. The open and closed circles represent unmethylated or methylated cytosines, respectively.

For assessing the methylation profile of CpG sites in Region-1, bisulfite genomic sequencing in various cancer cell lines was carried out. In Fig. 5A, the genomic sequence of OCTN2 promoter was displayed and CpG sites were marked. One methylated sequence of *OCTN2* spanning −325 to −92 bp in HepG2 cells was checked for alignment using MethBLAST and is presented in Fig. 5B. Other methylated sequences from LS174T, QBC-939 and U251 cells are presented in Fig S1, S2 and S3. In Fig. 5C, the methylation profiles showed that the rates of methylated CpG sites (mCpG/total CpG number) were 72.2% and 60.0% in HepG2 and LS174T cells, respectively, which is much higher than methylation rates measured in QBC-939 (20.0%) and U251 (11.1%) cells. Moreover, after 1 μM DCA treatment in HepG2, the rates of methylated CpG sites decreased to 13.3% (Fig. 6). Since the hypermethylated CpG sites within OCTN2 are rescued by DCA treatment in HepG2 and LS174T cells, these findings provide direct evidence that individual methylated CpG sites in the promoter may precisely regulate the expression of OCTN2.

### Effects of DCA on Enhancing Sensitivity of Cancer Cells to Oxaliplatin

**Figure 7:**
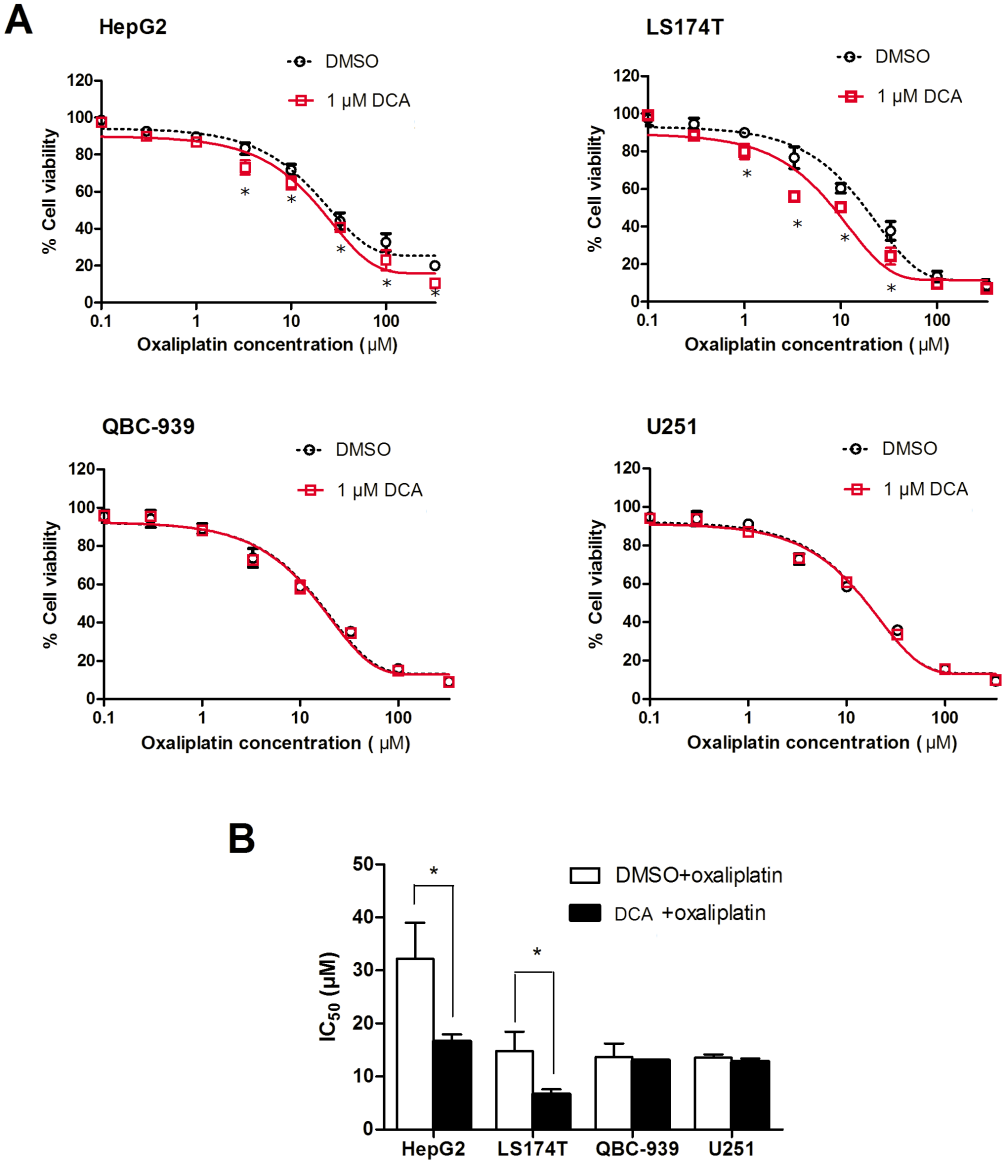
Demethylation of OCTN2 by DCA influenced cell viability to oxaliplatin. Cancer cells were pre-treated with 1 μM DCA for 1 week, seeded in 96-well plates at 1×10^4^ cells/well in 100 μl DMEM and 10% FBS. After 8 h, the cells were exposed to oxaliplatin at various concentrations (0.1, 0.3, 1.0, 3.3, 10, 33, 100 or 330 μM) for 48 h in fresh culture medium. Cell viability was determined by a MTS cell proliferating assay kit. Each concentration was determined in three wells. Data presented represent the mean ± S.D. of three independent experiments. (A), the cell viability at each concentration was displayed. Significant differences from DMSO treatment are denoted with an asterisk (*, *p*<0.05). (B), IC_50_ values were calculated by SPSS 13.0 software. Data presented represent the mean ± S.D. of three independent experiments. Significant differences from non-treated cells are denoted with an asterisk (*, *p*<0.05).

To further understand if methylation status of the *OCTN2* gene affects the sensitivity of cancer cells to oxaliplatin, cancer cells were pre-treated with DCA followed by exposure to oxaliplatin. Cell viability was assessed and the IC_50_ values were calculated. In Fig 7. A, when cells were exposed to oxaliplatin at 3, 10, 33 and 100 μM, cell viability of DCA-treated HepG2 and LS174T was decreased compared to DMSO-treated cells (*p*<0.05), but did not change in U251 and QBC-939 cells. In Fig. 7B, the IC_50_ values for DCA-treated HepG2 and LS174T cells were decreased to 51.6% and 44.8% (*p*<0.05), respectively, compared to DMSO-treated cells, but were unchanged in QBC-939 and U251 cells. Therefore, it is possible that DCA sensitizes HepG2 and LS174T cells to oxaliplatin through increasing OCTN2 expression and enhancing the uptake of oxaliplatin.

## Discussion

In this study, we found that hypermethylation within the *OCTN2* promoter is responsible for the down-regulation of *OCTN2* in HepG2 and LS174T cells. We precisely detected the degree of methylation of individual methylated CpG sites in the promoter region by MSP and BSP, which was inversely correlated with the expression of OCTN2 in different cancer cells. Moreover, DCA pre-treatment of HepG2 and LS174T cells increased oxaliplatin-induced cell death via OCTN2 demethylation.

The essential role of DNA methylation has attracted much interest as a possible mechanism underlying the aberrant expression of oncogenes, tumor suppressor genes and DNA repair genes in carcinoma cells. Recent findings show a role for DNA methylation in regulating the expression of transporters, such as SLC22A1, A2, A3 (OCT1, OCT2, OCT3), SLC22A18, SLC5A8, Ntcp, Oatp1b2, Bsep, ABCG2, ABCG5/G8 [22]–[27]. To understand the mechanism by which OCTN2 expression is repressed in HepG2 and LS174T cells, we treated these cell lines with the demethylating reagent DCA and observed an increase in OCTN2 mRNA and protein levels in HepG2 and LS174T cells, while no change occurred in QBC-939 and U251 cells. A recent study also found that the low levels of OCTN2 were increased by DCA in human papillomavirus16 E6- and E7- expressing keratinocytes [17]. DCA inhibits DNA methyltransferase enzymes (DNMTs) and further corrupts the integrity of the methylation imprint, thereby leading to the disruption of epigenetic signatures and regulation of gene expression [28]. Thus, these findings suggest that the down regulation of OCTN2 in HepG2 and LS174T cells may be due to aberrant promoter methylation.

Analyzing the three putative CpG islands within *OCTN2*, we observed that CpG islands displayed different methylation statuses in different cancer cells, and that hypermethylation could be reduced by DCA. Therefore, we sought to identify which CpG island plays a pivotal role in the regulation of OCTN2 expression. In the luciferase reporter assay, only the promoter Region-1 spanning −354 to +85 bp showed increased promoter activity. Moreover, methylation of Region-1 that mimics the methylation patterns in cells, completely inhibited the promoter activity. This result provides the evidence that Region-1 is a possible determinant of OCTN2 expression, because hypermethylation of Region-1 inhibited transcriptional activity of OCTN2 in LS174T and HepG2 cells.

Bisulfite genomic sequencing precisely detected the individual methylated CpG sites within promoter Region-1, which was significantly hypermethylated in HepG2 and LS174T compared to QBC-939 and U251 cells. The degree of DNA methylation was inversely correlated with the expression of OCTN2 in these cancer cells. These findings may reasonably explain the lower levels of OCTN2 in HepG2 and LS174T cells, compared to the expression in QBC-939 and U251 cells. Moreover, DCA reversed the methylation of CpG sites in Region-1 in HepG2 cells consistent with the increase in OCTN2 expression.

Methylated CpG dinucleotides within promoter Region-1 inhibit the expression of *OCTN2* probably through two mechanisms. First, many transcription factor bindings may be blocked by methylated CpG dinucleotides within the *OCTN2* promoter. We analyzed promoter Region-1 of *OCTN2* by TFSEARCH software (http://www.cbrc.jp/research/db/TFSEARCH.html), and found that the consensus binding sites of transcription factor specificity protein 1 (Sp1) and myeloid zinc finger 1 (MZF-1) overlapped some key CpG sites (Fig S4). Further work is in progress to investigate whether the methylation of specific CpG sites blocks these transcription factors. A second possible mechanism is the aberrant regulation of DNA methyltransferase enzymes and methylation-dependent recruitment of nucleoprotein factors such as the methy-CpG binding protein MeCP1 and MeCP2 [29]–[31]. We will investigate how the hypermethylated CpG sites of OCTN2 recruit these methylation-related proteins and repress the expression of OCTN2.

Given the essential role of OCTN2 in cancer cell uptake and thus treatment efficacy of oxaliplatin, methylation of *OCTN2* might be used as a target for enhancing therapeutic efficacy. After treatment with 1 μM DCA, HepG2 and LS174T cells became more sensitive to oxaliplatin, but not QBC-939 and U251 cells. Moreover, in DCA treated cells, the increased expression of OCTN2 conferred greater oxaliplatin uptake and more cytotoxicity than non-DCA treated cells. Although DCA in high concentrations causes global genomic instability and cytotoxicity, DCA in low concentrations acts mainly to inhibit DNMT rather than to induce cell death [32]. Thus, DCA was concomitantly administered with oxaliplatin, and the resulting restoration of OCTN2 expression enhanced the uptake and efficacy of oxaliplatin.

## Conclusion

In conclusion, we revealed the epigenetic mechanism of the down-regulation of *OCTN2* in different cancer cells. The promoter region spanning −354 to +85 was a determinant of OCTN2 expression. The degree of methylated CpG sites within the region was inversely correlated with the levels of OCTN2 in cancer cells. Application of the demethylating agent, DCA, reversed the hypermethylation status of the *OCTN2* promoter and increased OCTN2 expression, thereby leading to enhanced uptake of oxaliplatin-causing cancer cells to be more sensitive to oxaliplatin. Given the essential role of OCTN2 in cancer cell uptake and thus treatment efficacy of several chemotherapeutics, the pretreatment of a demethylating reagent is a possible strategy for optimization of pharmacotherapy against cancers.

## Supporting Information

Figure S1 Methylation profiles around the transcriptional start site of *OCTN2* in LS174T cells. DNA was extracted and subjected to bisulfite modification. Bisulfite modified DNA was amplified by BSP and sequenced as described under *Materials and methods*. Pointed star represents methylated CpG sites.
(TIF)

Figure S2 Methylation profiles around the transcriptional start site of *OCTN2* in QBC-939 cells. DNA was extracted and subjected to bisulfite modification. Bisulfite modified DNA was amplified by BSP and sequenced as described under *Materials and methods*. Pointed star represents methylated CpG sites.
(TIF)

Figure S3 Methylation profiles around the transcriptional start site of *OCTN2* in U251 cells. DNA was extracted and subjected to bisulfite modification. Bisulfite modified DNA was amplified by BSP and sequenced as described under *Materials and methods*. Pointed star represents methylated CpG sites.
(TIF)

Figure S4 Transcription factor binding site analysis of the promoter region of *OCTN2* by TFsearch software. To reveal the mechanism by where methylated CpG sites inhibit *OCTN2* transcription, we mapped the putative consensus sequences for the transcription factors. CpG sites are on the red lines. Putative consensus sequences are indicated by dotted lines.
(TIF)

Table S1 Primers for Quantitative RT-PCR, MSP and BSP.
(PDF)

Table S2 Primers for the construction of luciferase reporter vectors.
(PDF)
