# Supplementary figures and images for: Different Involvement of Promoter Methylation in the Expression of Organic Cation/Carnitine Transporter 2 (OCTN2) in Cancer Cell Lines
Source: PLoS One. 2013 Oct 16;8(10):e76474. doi: 10.1371/journal.pone.0076474 (PMC3797819; doi:10.1371/journal.pone.0076474)

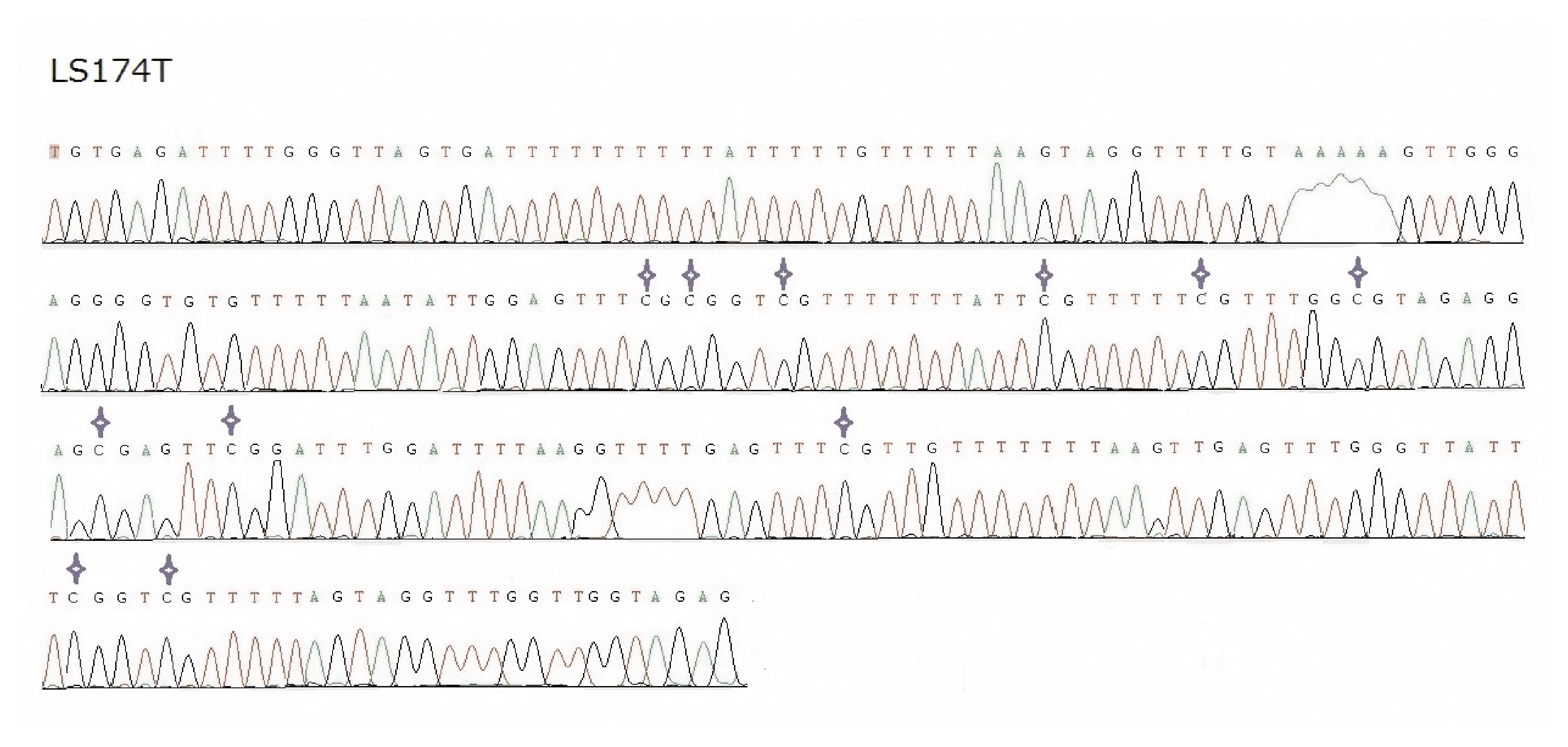

Supplement: Figure S1 — Methylation profiles around the transcriptional start site of OCTN2 in LS174T cells. DNA was extracted and subjected to bisulfite modification. Bisulfite modified DNA was amplified by BSP and sequenced as described under Materials and methods. Pointed star represents methylated CpG sites. (TIF) [file pone.0076474.s001.tif]

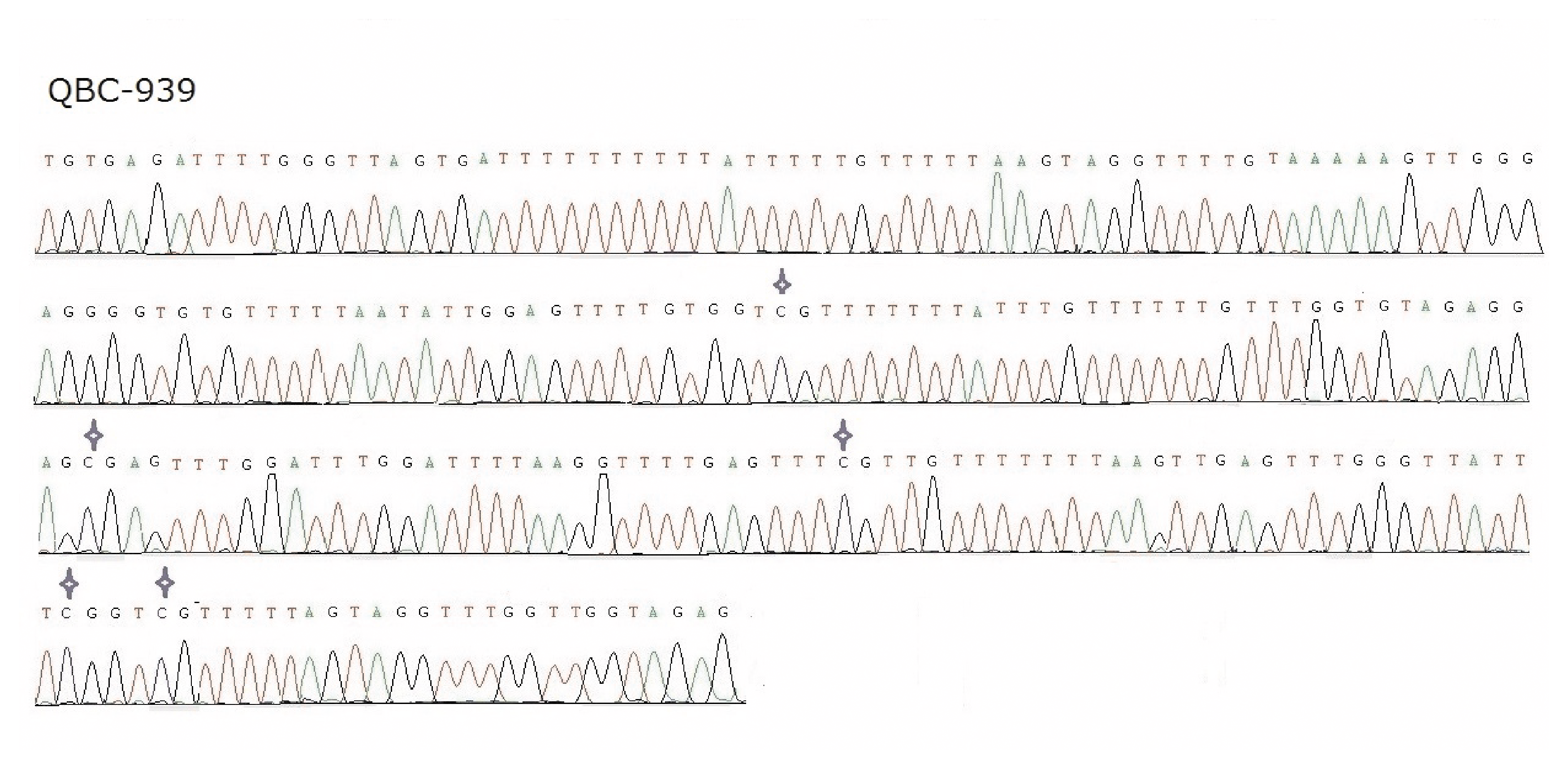

Supplement: Figure S2 — Methylation profiles around the transcriptional start site of OCTN2 in QBC-939 cells. DNA was extracted and subjected to bisulfite modification. Bisulfite modified DNA was amplified by BSP and sequenced as described under Materials and methods. Pointed star represents methylated CpG sites. (TIF) [file pone.0076474.s002.tif]

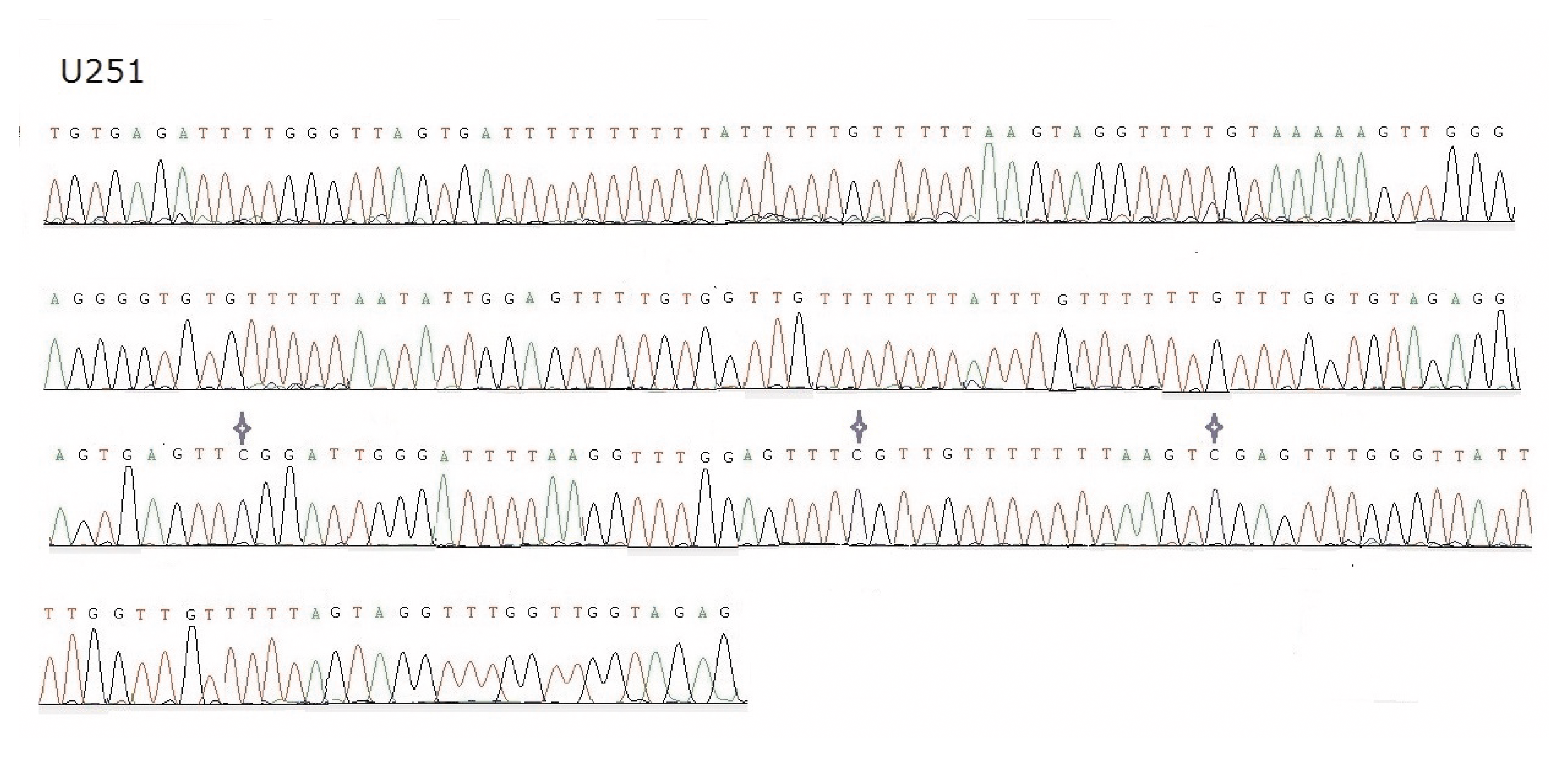

Supplement: Figure S3 — Methylation profiles around the transcriptional start site of OCTN2 in U251 cells. DNA was extracted and subjected to bisulfite modification. Bisulfite modified DNA was amplified by BSP and sequenced as described under Materials and methods. Pointed star represents methylated CpG sites. (TIF) [file pone.0076474.s003.tif]

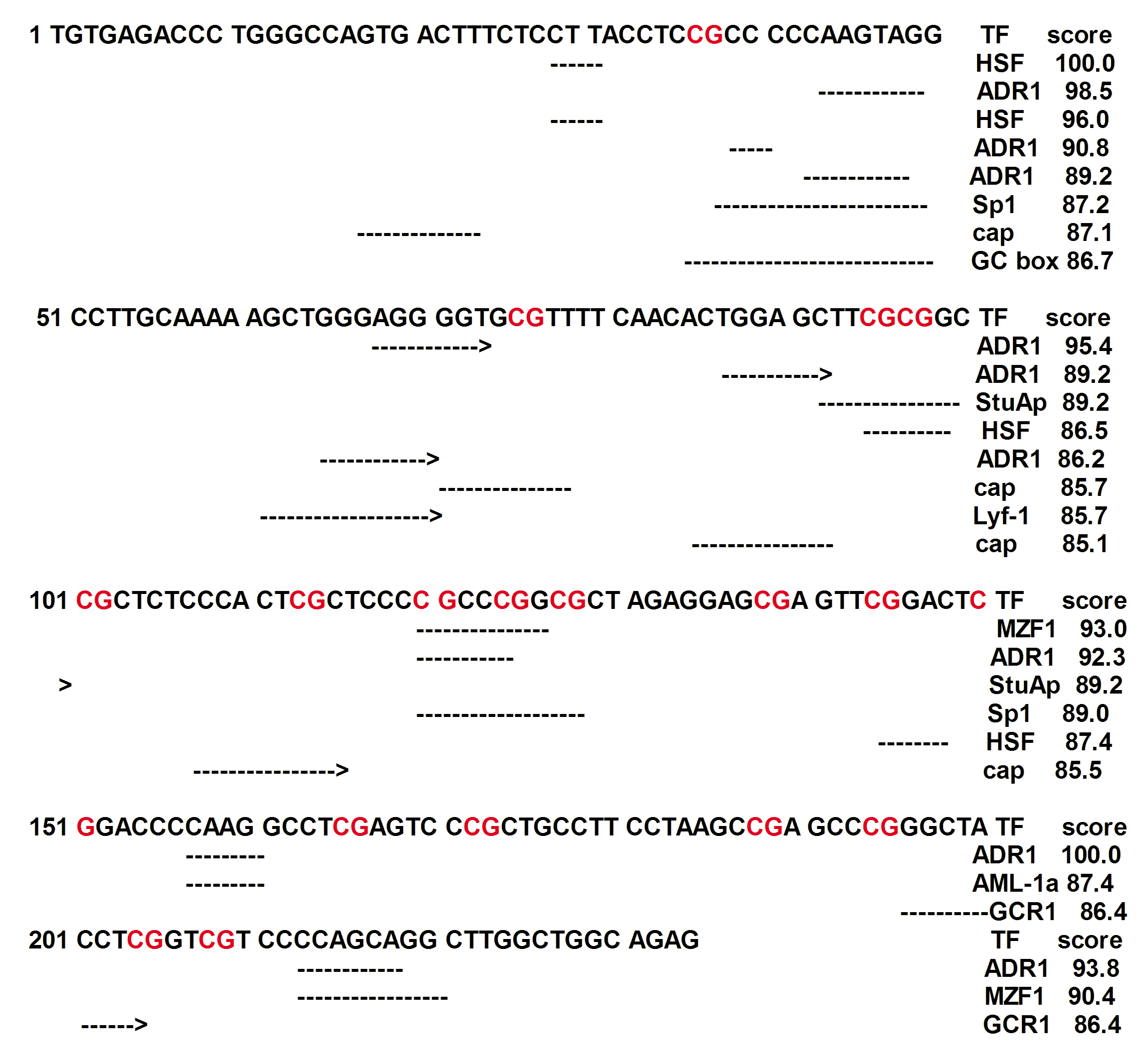

Supplement: Figure S4 — Transcription factor binding site analysis of the promoter region of OCTN2 by TFsearch software. To reveal the mechanism by where methylated CpG sites inhibit OCTN2 transcription, we mapped the putative consensus sequences for the transcription factors. CpG sites are on the red lines. Putative consensus sequences are indicated by dotted lines. (TIF) [file pone.0076474.s004.tif]
