# Supplementary material for: Different Involvement of Promoter Methylation in the Expression of Organic Cation/Carnitine Transporter 2 (OCTN2) in Cancer Cell Lines
Source: PLoS One. 2013 Oct 16;8(10):e76474. doi: 10.1371/journal.pone.0076474 (PMC3797819; doi:10.1371/journal.pone.0076474)
Supplement: Table S1 — Primers for Quantitative RT-PCR, MSP and BSP. (PDF) [file pone.0076474.s005.pdf]

Table.S1. Primers for Quantitative RT-PCR, MSP and BSP.

Name	Primers	Product length	Tm
RT-*OCTN2*	F: 5’-CCATTGTGACCGAGTGGAACC-3’<br>R: 5’-ACATTCTTCCGGCCAAACCTG-3’	136 bp	60 ℃
RT-*β-actin*	F: 5’-TTGCCGACAGGTGCAGAAGGA-3’<br>R: 5’- AGGTGGACAGCGAGGCCAGGAT-3’	128 bp	60 ℃
MSP-CpG1 M primer	F:5’-CTACCAACCAAACCTACTAAAAACG-3’<br>R:5’-TAATATTGGAGTTTCGCGGTC-3’	152 bp	60 ℃
MSP-CpG1 U primer	F:5’-AATATTGGAGTTTTGTGGTTGT-3’<br>R:5’-TCTACCAACCAAACCTACTAAAAACA-3’	152 bp	60 ℃
MSP-CpG2 M primer	F:5’-AACCATTAAAAATAATACTAACGCT-3’<br>R:5’-TTGTTGTTTGGTTTGTTTGGTC-3’	205 bp	62 ℃
MSP-CpG2 U primer	F:5’-AACCATTAAAAATAATACTAACACT-3’<br>R:5’-GTTGTTTGGTTTGTTTGGTTG-3’	203 bp	62 ℃
MSP-CpG3 M primer	F:5’-TTTGTTATTAGGTTGGAGCGTAGTC-3’<br>R:5’-AATCACGAAATCAAAAAATCGAA-3’	226 bp	60 ℃
MSP-CpG3 U primer	F:5’-TGTTATTAGGTTGGAGTGTAGTTGT-3’<br>R:5’-CAAATCACAAAATCAAAAAATCAAA-3’	224 bp	60 ℃
BSP-CpG1	F:5’- TGTGAGATTTTGGGTTAGTGATTT-3’<br>R:5’-CTCTACCAACCAAACCTACTAAAAAC-3’	234 bp	58 ℃
