# Supplementary material for: Different Involvement of Promoter Methylation in the Expression of Organic Cation/Carnitine Transporter 2 (OCTN2) in Cancer Cell Lines
Source: PLoS One. 2013 Oct 16;8(10):e76474. doi: 10.1371/journal.pone.0076474 (PMC3797819; doi:10.1371/journal.pone.0076474)
Supplement: Table S2 — Primers for the construction of luciferase reporter vectors. (PDF) [file pone.0076474.s006.pdf]

Table. S2. Primers for the construction of Luciferase reporter vector.

Name	Primers	Product length
CpG1	F : 5' -CCGCTCGAGAAGATTAGGCGGTGTCAAGAGC-3<br>R: 5'- GGGGTACCACATAGGGCGCACGACCAG-3'	439 bp
CpG2	F: 5' -CCGCTCGAGGTCTTGGGTCGCCTGCTGC 3'<br>R: 5'- GGGGTACCGGTGCTCCGGGGTCGCTAT 3'	262 bp
CpG3	F: 5' -CCGCTCGAGCCCTGTAAGTAATTGTTTGC 3'<br>R: 5' - GGGGTACCTCACCCTGAACCCATCTT 3'	404 bp

Forward primer 5’ underline is indicated as Xho I, and reversed primer 5’ underline is indicated as Kpn I.
